# Genome-Wide Profiling of the Microrna Transcriptome Regulatory Network to Identify Putative Candidate Genes Associated with Backfat Deposition in Pigs

**DOI:** 10.3390/ani9060313

**Published:** 2019-06-01

**Authors:** Xin Liu, Jianfei Gong, Ligang Wang, Xinhua Hou, Hongmei Gao, Hua Yan, Fuping Zhao, Longchao Zhang, Lixian Wang

**Affiliations:** 1 Institute of Animal Science, Chinese Academy of Agricultural Sciences, Beijing 100193, China; firstliuxin@163.com (X.L.); Gjf18553676192@163.com (J.G.); ligwang@126.com (L.W.); 7hxh73@163.com (X.H.); gaohongmei_123@126.com (H.G.); zcyyh@126.com (H.Y.); zhaofuping@caas.cn (F.Z.); 2 College of Animal Science and Technology, Qingdao Agricultural University, Qingdao 266109, China

**Keywords:** backfat deposition, candidate gene, expression profile, pig, regulatory network

## Abstract

**Simple Summary:**

Backfat thickness is an important characteristic in pig breeding. In this study, the key microRNAs (miRNAs) and genes associated with pig backfat deposition were detected and characterized using RNA sequencing between adipose tissues of high-backfat and low-backfat pigs. Strong candidate mRNA‒miRNA interaction pairs were identified to affect backfat deposition through the regulation of target genes by miRNAs. These results provide novel insights into the backfat deposition mechanism in pigs.

**Abstract:**

Backfat deposition is strongly related to carcass traits, growth rate, feed conversion rate, and reproductive performance in pig production. To understand the molecular mechanisms underlying porcine backfat thickness phenotypes, transcriptome and miRNA profiling of backfat from high-backfat thickness and low-backfat thickness pigs were performed by RNA sequencing. Twenty genes encoding for miRNAs and 126 genes encoding for protein-coding genes were found to be differentially expressed between the two libraries. After integrative analysis of DEMs targets and DEGs, a total of 33 mRNA‒miRNA interaction pairs were identified, and the regulatory networks of these pairs were determined. Among these genes, five (*AQP9*, *DKK3*, *GLYCTK*, *GLIPR1*, and *DUSP2*) related to fat deposition were found to be strong candidate genes, and mir-31-5p/*AQP9* and mir-31-5p/*GLIPR1* may play important roles in fat deposition. Additionally, potential adipogenesis-related genes and miRNAs were identified. These findings improve the current understanding of the molecular genetic mechanisms of subcutaneous fat deposition in pigs and provide a foundation for further studies.

## 1. Introduction

Backfat deposition is an important characteristic studied in pigs because of its strong relationship with carcass traits, growth rate, feed conversion rate, and reproductive performance in pig production. Additionally, backfat deposition and fat traits affect the human nutritional value of pig products [1]. Reducing backfat thickness is a major goal in swine breeding programs to provide lean meat to consumers. In addition, pigs are more closely related to humans in terms of anatomy, genetics, and physiology than mice, and thus pigs are more suitable for human health research [2]. Examining adipogenesis in pigs can provide insight into human obesity, diabetes, and even cardiovascular disease.

MicroRNA (miRNA) is defined as a series of 18‒24 nucleotides non-coding fragments that negatively regulate target gene expression and cause translational repression or degradation and deadenylation of target genes by binding to the 3′ untranslated region of complementary target genes [3,4]. MicroRNAs have been demonstrated to play a regulatory function in wide-ranging biological processes, for example, metabolism, pathogenesis, development, differentiation, and lipogenesis, among other processes [5,6]. The first reported effect of miRNAs on lipid metabolism was in *Drosophila*, in which the absence of miR-14 resulted in an increase in triacylglycerol and diacylglycerol accumulation [7]. Studies of miRNAs focusing on lipogenesis showed that some miRNAs were associated with adipocyte differentiation, adipose tissue morphology, adipocyte size, and metabolic parameters [8,9,10,11]. To date, only 430 mature miRNA sequences in pigs have been included in the miRBase lists (http://www.mirbase.org) [12], and few studies have described the roles of miRNAs in porcine lipogenesis during development and in different breeds [13,14,15,16]. The transcriptome shows RNAs transcribed in different tissues as well as spatial and temporal expression characteristics [17]. The RNA-seq approach is used to compare the transcription profile and identify differentially expressed genes (DEGs) [18,19]. Transcriptome analysis of lipogenesis in pigs has been conducted for different breeds, developmental periods, and fat deposition stages. Additionally, many genes that function in lipid deposition and metabolic pathways have been identified [1,20]. However, while many related genes were identified through transcriptome analysis, the mechanisms by which these genes affect the adipose deposition were still unknown.

In this study, miRNA sequencing and mRNA sequencing were performed to identify differentially expressed miRNAs (DEMs) and differentially expressed genes (DEGs), respectively, in adipose tissue from three pairs of full siblings (full-sib) with high-backfat thickness (HBFT) and low-backfat thickness (LBFT) individuals in each pair. Next, miRNA-seq and mRNA-seq were integrated to identify potential key genes and reveal the mechanism of fat deposition.

## 2. Material and Methods

### 2.1. Ethics Statements

All methods and procedures in the study were carried out according to the standard guidelines on experimental animals (No. IASCAAS-AE-09), which were established by the Animal Ethics Committee of the Institute of Animal Science, Chinese Academy of Agricultural Sciences (IAS-CAAS) (Beijing, China). The experimental protocols were approved by the Science Research Department of IAS-CAAS (Beijing, China). 

### 2.2. Animals and Sample Collection

**Figure 1:**
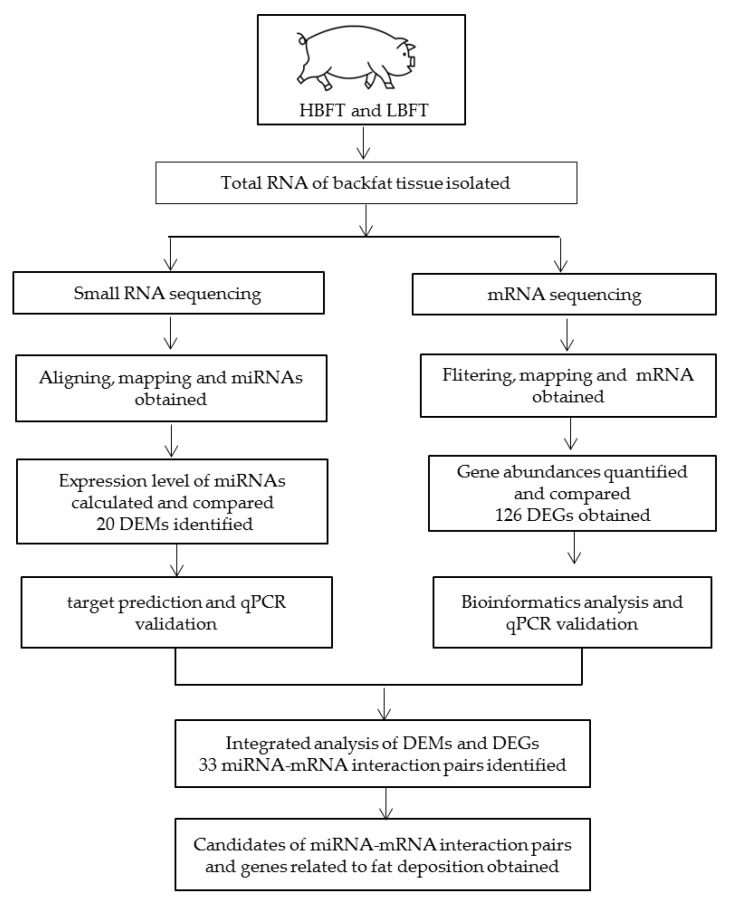
Workflow of the study with the experimental design and main results.

The Large White × Minzhu F2 resource population, which has been described previously [21], was used. The pigs were reared at the pig farm of the Institute of Animal Science, CAAS, with the same nutritional and environmental conditions. Pigs were weighed and sacrificed at 240 ± 7 days in a commercial abattoir following standard commercial procedures. Samples of adipose tissue from the backfat of pigs were submerged in liquid nitrogen and stored at ‒80 °C in a refrigerator until use. Backfat thickness (6–7 ribs) (BFT) was measured using Vernier calipers. Based on the phenotypic data of the population, two phenotypic groups, each containing three pigs with HBFT or LBFT, were assembled. One HBFT pig and one LBFT pig were full-sib, and thus three full-sibs were selected. Additionally, the three pairs of test pigs were born by three pairs of unrelated boars and sows. Individual phenotypic data are shown in Table 1. The workflow of the study with the experimental design has been shown in Figure 1.

### 2.3. Small RNA Library Construction and Sequencing

Total RNA of backfat tissue was isolated using TRIzol reagent (Invitrogen, Carlsbad, CA, USA). Adequate quality and quantity of RNA were ensured by using a Bioanalyzer 2100 (Agilent Technologies, Santa Clara, CA, USA). Eighteen- to 30-nucleotide (nt) RNA fragments were enriched by polyacrylamide gel electrophoresis. First, 3′ adapters were added and the 36–44-nt RNAs were enriched. Next, 5′ adapters were ligated to the RNAs as well. The finished products were amplified by reverse transcription (RT)-PCR. A cDNA library was constructed by PCR products (140–160 base pairs, bp), and they were sequenced using an Illumina HiSeq 2500 (Illumina, San Diego, CA, USA). 

### 2.4. Alignment and Identification of Small RNA

The raw reads, which included adapters or low-quality bases, may affect the sequence assembly and analysis. The raw data on miRNAs sequencing have been uploaded to the NCBI database (SRR7892706, SRR7892707, SRR7892708, SRR7892709, SRR7892710, and SRR7892711). Thus, raw reads require further filtering to obtain clean tags following the following rules: low-quality reads containing more than one low-quality (Q-value ≤ 20) base or unknown nucleotides were removed, and reads without 3′ adapters or 5′ adapters, containing 3′ and 5′ adapters but no small RNA fragment between them, containing polyA in small RNA fragment, or shorter than 18 nt (not including adapters), were removed. All clean tags were aligned with small RNAs in the GenBank database (Release 209.0) and Rfam database (11.0) to identify and remove rRNA, scRNA, snoRNA, snRNA, and tRNA. All clean tags were also aligned with the reference genome to remove the tags mapped to exons, introns, or repeat sequences. Next, the filtered tags were searched against the miRBase database (Release 21) to identify known porcine miRNAs. The unannotated tags were predicted and identified as a novel miRNA by using Mireap_v0.2 software according to the tags’ positions in the genome and their hairpin structures.

### 2.5. DEMs Analysis and Target Gene Prediction

The miRNA expression level was calculated and normalized to transcripts per million (TPM). miRNAs with a fold change ≥ 2 and *p* value < 0.05 by comparison of each full-sib pair (L1‒H1, L2‒H2, L3‒H3) were identified as significant DEMs, then three sets of intersecting DEMs as the candidates were further studied. Three bioinformatics tools for miRNA target prediction were used to predict targets, namely, RNAhybrid (v2.1.2) +sum_light (v6.01), Miranda (v3.3a), and TargetScan (v7.0) [22]. 

### 2.6. mRNA Sequencing and Data Analysis

Total RNA was isolated using TRIzol reagent (Invitrogen), following the manufacturer’s instructions. The quality and quantity of the RNA samples were detected by a RNA 6000 Nano LabChip Kit and a 2100 Bioanalyzer (Agilent Technologies). The mRNA was fragmented into short fragments and reverse-transcribed into cDNA with random primers. Second-strand cDNA were synthesized by DNA polymerase I, RNase H, dNTP. Then, the cDNA fragments were purified with a QiaQuick PCR purification kit (QIAGEN, Hilden, Germany). The adapters and Poly(A) were added, the ligation products were sequenced using an Illumina HiSeq^TM^ 2500 by Gene Denovo Biotechnology Co. (Guangzhou, China), and 125 bp paired-end reads were generated. The data of mRNA sequencing have been uploaded to the NCBI database (SRR7892549, SRR7892550, SRR7892551, SRR7892552, SRR7892553, and SRR7892554). Raw reads were filtered to remove adapters, more than 50% of low-quality (Q-value ≤ 20) bases, and more than 10% unknown nucleotides. The short read was mapped to the ribosome RNA (rRNA) database by using the alignment tool Bowtie2. The reads with removed rRNA reads were then mapped to the Sus scrofa 10.2 reference genome by TopHat2 (version 2.0.3.12). Gene abundances were quantified by RSEM software, and the gene expression level was normalized by using the fragments per kilobase of transcript per million (FPKM) mapped reads method. 

### 2.7. DEGs Analysis and Bioinformatics Analysis

To identify differentially expressed genes across samples, the edgeR package in R software (http://www.r-project) was used [23]. The significant DEGs from each full-sib pair (L1‒H1, L2‒H2, and L3‒H3) of comparison were identified with a |fold-change| ≥ 2 and false discovery rate (FDR) < 0.05, and three sets of intersecting DEGs were further studied as candidates. Candidate DEGs were then subjected to enrichment analysis of GO functions and the KEGG pathway, which were performed in the Integrated Discovery (DAVID) website (http://david.abcc.ncifcrf.gov/) [24]. GO was used to classify gene functions in terms of three aspects: molecular function, cellular components, and the biological process. Because of the interaction of genes in certain biological functions, pathway-based analysis was used to further understand the gene biological function. As the major public pathway database, KEGG identified a significantly enriched metabolic analysis and regulatory network in DEGs compared with the whole genome background.

### 2.8. Validation of RNA-Seq Data by q-PCR

Quantitative real-time PCR was used to confirm the RNA-seq results for miRNA and mRNA transcripts. Q-PCR amplification was performed using SYBR^®^ Select Master Mix (ABI part number 4472908) in an ABI 7900HT instrument (Applied Biosystems, Foster City, CA, USA). The cDNA of six samples (three HBFT and three LBFT) was used as templates for qPCR. The mRNA primers and miRNA-specific forward primers are shown in Table S1. The qPCR reaction conditions were as follows: 95 °C for 15 min, followed by 40 cycles of denaturation at 95 °C for 10 s and renaturation at 60 °C for 30 s. U6 and GAPDH were used as internal controls to correct for miRNA and mRNA analytical variations, respectively. The expression levels were calculated using the delta‒delta Ct method (2^−ΔΔCT^). A t-test was performed to evaluate the statistically significant of expression difference between HBFT and LBFT. Then, the trend of gene differential expression between HBFT and LBFT was consistent or not in RNA-seq results and qPCR results.

## 3. Results

### 3.1. Mapping Small RNA Reads and Sequencing Analysis

**Figure 2:**
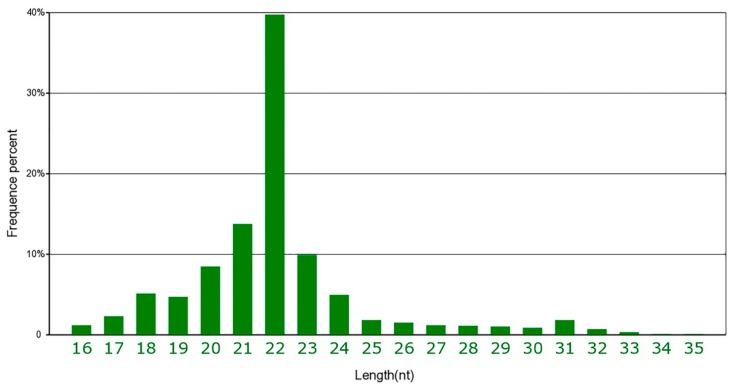
The distribution of small RNA reads.

The genome-wide miRNA expression of HBFT and LBFT pigs was analyzed by small-RNA sequencing to determine the molecular mechanisms of fat deposition. A total of approximately 78477,552 raw reads were obtained from the six sRNA libraries. After eliminating adaptor sequences and low-quality reads, clean reads were retained, which corresponded to an average of 84.47% of the total reads. The summary of small RNA sequencing data is shown in Table S2. As shown in Figure 2, most sequences were 20–23 nt in length with a peak at 22 nt, representing mature miRNA; the remaining small RNAs included rRNA, scRNA, snRNA, snoRNA, tRNA, and others. All clean reads were searched against the miRBase database (Release 21) to identify porcine miRNAs, and a total of 309 mature porcine miRNAs were annotated in the miRBase database. The homology search detected 796 known miRNAs and 166 novel miRNAs in the six sRNA libraries.

### 3.2. miRNA Expression Profiling and Differential Expression

**Figure 3:**
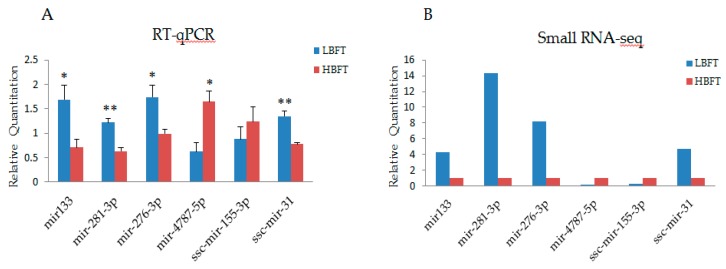
Validation of small RNA-sequencing data using reverse transcription real-time quantitative polymerase chain reaction (RT-qPCR) (**A**), RT-qPCR results of six selected miRNAs (**B**), and small RNA-seq results of six microRNAs (miRNAs) (Y-axis represents that the transcripts per million (TPM) of each gene in low-backfat thickness (LBFT) and high-backfat thickness (HBFT) groups was normalized by TPM of LBFT).

To identify miRNAs related to differences in backfat thickness between HBFT and LBFT pigs, DEMs of each full-sib pairwise comparisons were performed (>2-fold change, *p* < 0.05) and intersecting DEMs of three pairs were selected as candidates. Because full-sibs were used in this experiment and had basically the same genetic background, this analysis method could reduce the false positive rate and detect the DEMs more accurately. Compared to the LBFT library, 20 miRNAs were differentially expressed, including five upregulated and 15 downregulated miRNAs (shown in Table S3). To validate the miRNA identified by sequencing, six representative miRNAs were analyzed by stem-loop quantitative real-time PCR (qPCR) in HBFT and LBFT pigs. The qPCR results from downregulated miRNAs were consistent with the results of sequencing, and two upregulated miRNAs from sequencing data were consistently increased based on the qPCR results (Figure 3). 

### 3.3. RNA Sequencing Analysis and DEGs Detected

RNA sequencing (RNA-seq) was performed to identify the genes differentially expressed in the adipose transcriptomes of HBFT and LBFT pigs. A total of approximately 603242,210 raw reads was obtained from the six sRNA libraries. After quality trimming, on average, 87449,408 clean reads were generated from each sample. After ribosome reads were removed, the data were compared with the reference genome (Sus scrofa 10.2), and the average mapping rate was 74.25%. A summary of the data is given in Table S4.

**Figure 4:**
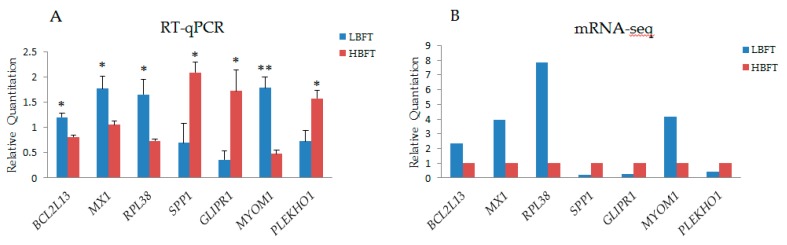
Validation of mRNA-seq data using RT-qPCR (**A**), RT-qPCR results of seven selected miRNAs (**B**), and mRNA-seq results of seven genes (Y-axis represents that the fragments per kilobase of transcript per million (FPKM) of each gene in LBFT, and HBFT groups were normalized by FPKM of LBFT).

DEGs between the adipose tissue of HBFT and LBFT pigs were detected after the basic analysis of sequencing results, using the same analysis strategy as overall DEMs presented above, and 126 DEGs were detected, including 71 upregulated genes and 55 downregulated genes (>2-fold difference, false discovery rate < 0.05) (shown in Table S5). The heat map of DEGs was shown in Figure S1. The function and related miRNAs of candidate DEGs, which have been reported with the functions related to fat synthesis, deposition, and metabolism, are listed in Table 2, and the detailed descriptions and related reports of some candidates are shown in the discussion section. The deep sequencing findings were verified by q-PCR in pigs with extreme backfat thickness phenotypes. Seven DEGs previously reported to be directly or indirectly associated with backfat thickness or over-expressed with a high fold-change were selected for quantitative verification, and the results were consistent with those of deep sequencing in terms of the direction of regulation and the statistical significance (Figure 4). 

### 3.4. Integrated Analysis of miRNA Sequencing and RNA-Seq

**Figure 5:**
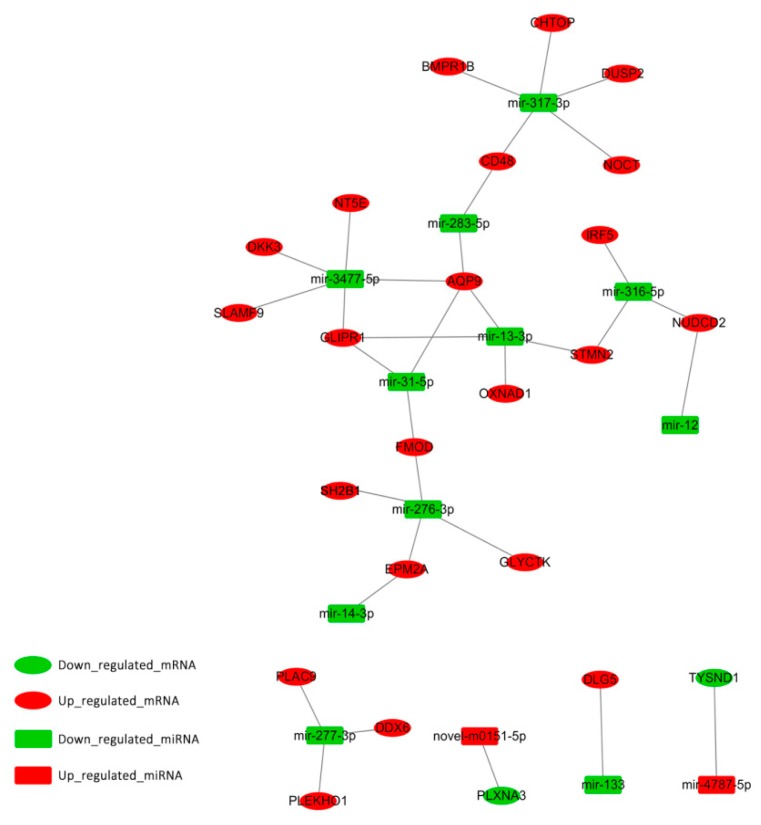
Regulatory network of significantly differentially expressed miRNA-mRNA pairs between HBFT and LBFT pigs. Up_regulated_mRNA (down-regulated mRNA) means that the gene is highly (low) expressed in the HBFT group and low (highly) expressed in the LBFT group. Up_regulated_miRNA (down-regulated miRNA) means that the miRNA is highly (low) expressed in the high backfat HBFT group and low (highly) expressed in the LBFT group.

To understand the biological significance of adipose-regulated miRNAs, their target mRNAs were predicted. RNAhybrid (v2.1.2) +sum_light (v6.01), Miranda (v3.3a), and TargetScan (v7.0) software were used to predict the biological target genes of each DEM. DEGs and target genes of DEMs were analyzed, and only miRNA‒DEGs pairs showing a negative correlation were selected. A total of 33 mRNA‒miRNA interactions were identified, including 23 annotated genes (Figure 5). Of the 23 annotated genes, the expression of 21 genes with 11 downregulated miRNAs was increased, while the expression of two genes with two upregulated miRNAs was decreased in HBFT pigs compared to in LBFT pigs. 

To understand the functional significance of genes with altered expression, GO and pathway analysis were performed. Among the 23 intersection genes, the enriched GO terms for genes were related to various biological regulations, cell parts, and bindings. Through pathway and gene function analysis, several genes were mapped into certain pathways associated with adipogenesis regulation. GLI pathogenesis-related 1 (*GLIPR1*) was in the peroxisome proliferator-activated receptor (PPARα) pathway. Dual-specificity phosphatase 2 (*DUSP2*) was associated with the MAPK pathway, and Dickkopf WNT signaling pathway inhibitor (*DKK3*) was included in the Wnt pathway. 

## 4. Discussion

In the present study, the systematic expression profiles of miRNAs and the transcriptome in adipose tissue were presented by deep sequencing in three full-sib pairs of pigs with extreme backfat thickness, selected from the Large White × Minzhu F2 resource population. Minzhu, an indigenous pig breed, lives in northeast China. Because of the cold environment, Minzhu has developed large fat deposits and high-backfat thickness [25]. The F2 population is a separate group, with some individuals showing parental characteristics. Full-sib pairs were used in this study to reduce the false-positive results caused by genetic background noise. Thus, full-sib pairs with extreme backfat thickness were chosen from the F2 population for this research. Deep sequencing has the advantage of high detection sensitivity and low noise, so it has been widely applied for analyzing globe gene expression patterns and identifying new transcripts. Thus, in this study, deep sequencing was used to identify the expression pattern of miRNAs and mRNAs in the backfat of test pigs. 

Significant advances have been made regarding the roles of miRNAs in the meat quality of pigs, particularly in skeletal muscle development, and a few studies have focused on backfat deposition [17,26,27,28]. In this study, some miRNAs were abundantly expressed in subcutaneous adipose tissue—for example, ssc-miR-10b, ssc-miR-26a, ssc-miR-22-3p, ssc-miR-143-3p, ssc-let-7, miR-143, and miR-26. Some of these miRNAs (e.g., miR-143, let-7, miR-148, and miR-26) have been shown to play an important regulatory role in fat development [14,29], and some new miRNAs related to backfat deposition were identified by deep sequencing technology. The DEMs detected between LEAN and FAT pigs were also reported in previous research, and some of the 31 detected DEMs were different from ours [29]. This difference may be caused by the experimental pigs; they used Italian Large White pigs in their experiment, while we used 240-day-old F2 (Large White × Minzhu) in our test. However, there were similar results for, for example, miR-133a, which was downregulated in adipose tissue [29]. Changes in five lipid-related DEMs (ssc-miR-31, miR-14-3p, miR-133, miR-281, and ssc-mir-155-3p) were identified by our deep sequencing. In our study, the expression of miR-31 was higher in LBFT compared to in HBFT, so miR-31 negatively regulated the backfat deposition of pigs. It also has been reported that miR-31 was downregulated during the adipogenic differentiation of adipose-derived stem cells [30]. miR-14 was another detected DEM found to regulate fat metabolism. The levels of triacylglycerol and diacylglycerol were increased by miR-14’s deletion, whereas the opposite effect was found for the increase of mir-14 copy number [7]. miR-133a, a muscle-related miRNA, plays a vital role in muscle development and function. miR-133a-deficient mice showed low exercise capacity and resting metabolic rate [31]. Additionally, miR-133a was differentially expressed in high- and low-abdominal fat chickens and was identified to be related to abdominal adipose tissue [32]. mir-281 was detected in the fat bodies of *Manduca sexta,* and was identified to be associated with the long-chain fatty acid elongase. Thus, mir-281 was involved in lipid metabolism [33]. In a study of obesity, miR-155 was found to be a promising target. The study showed that miR-155 deletion can restrict fat accumulation induced by a high-fat diet, because the deletion of miR-155 led to increased adipogenesis, insulin sensitivity, and energy uncoupling; meanwhile, inflammation in white adipose was limited. miR-155 was identified as a novel candidate target for improving obesity resistance [34]. The other DEMs detected in this study may be new candidate miRNAs for the regulation of adipogenesis.

The profile of the subcutaneous adipose tissue transcriptome in pigs was delineated by deep sequencing, and DEGs were detected between HBFT and LBFT pigs in the comparative analysis. We analyzed the biological functions of 126 differentially expressed genes using GO term enrichment. The analysis showed that these genes were mainly associated with the metabolic process, single-organism processes, protein binding, and glycerate kinase activity. More genes were enriched in general biological processes and basic functions. However, some of these genes may be strong candidates for regulating adipogenesis. Aquaporin 9 (*AQP9*) facilitated the uptake of glycerol in hepatic tissue. In recent studies, AQP9 was detected in adipocytes and was found to be associated with adipose tissue metabolism. The increased expression of AQP9 in subcutaneous adipose tissue was associated with human obesity [35,36]. *DKK3* is related to the Wnt/B-catenin signaling pathway, and related genes of the Wnt/B-catenin signaling pathway are expressed during adipose differentiation and development [37]. This signaling pathway can block the expression of key transcription factors (C/EBPα and PPARγ) of adipose differentiation, thus affecting adipose differentiation [38]. So, the *DKK3* gene may play a different role in adipose through this pathway. Studies of liver steatosis revealed that insulin sensitivity, enhanced by a high-fat diet or genetic defects, was improved by the specific overexpression of the DKK3 gene [39]. Glycerate kinase (*GLYCTK*) is related to glycerate kinase activity through GO annotations and enriches the glycerolipid metabolism pathway. Recent studies of GLYCTK have focused on D-glyceric aciduria. Mutations in *GLYCTK* caused deficiencies in D-glycerate kinase and D-glyceric aciduria [40]. GLIPR1 regulates lipid metabolism via PPARα. DUSP2, detected as DEG in our study, was reported to negatively regulate the mitogen-activated protein kinase family (MAP family), associated with cellular proliferation and differentiation and with the MAPK signaling pathway, which is an important pathway to inhibit adipogenesis [41]. Expect for these known lipid-related genes, the other DEGs detected in this study may be candidate genes for adipogenesis. After bioinformatics analysis, more function verification experiments will be needed to detect and prove their effects on fat.

In order to better explore the gene function associated with lipid metabolism and fat deposition, expression patterns of DEMs and DEGs were subjected to integrated analysis in our study and 33 mRNA-miRNA interactions were identified. In the integrated analysis, the miRNA‒gene regulatory pairs were detected by miRNA target gene prediction software, according to the principle of matching the seed sequences of miRNA with the 3’UTR region of the target gene, and met the requirement of negative correlation between the expression of miRNA and target gene. *GLIPR1* and *AQP9* were found to be regulated by mir-13-3p, mir-31-5p, and mir-3477-5p. Mir-3477-5p also regulated *DKK3*. These miRNAs may act on adipogenesis by regulating their target genes. These regulatory relationships may partly explain the mechanism of fat deposition, in particular, mir-31 regulated adipogenic differentiation, as described above. Thus, mir-31-5p/*AQP9* and mir-31-5p/*GLIPR1* may be strong candidates. In the following work, we will further verify the important candidate miRNA‒gene regulatory pairs because the target gene of miRNA in this study was predicted, so more evidence will be needed to determine their important functions. All of these interaction pairs were checked for potential previous validation by miRTarBase (http://mirtarbase.mbc.nctu.edu.tw/php/statistics.php), and, unfortunately, these pairs have not been validated. In the following work, we will further verify the important candidate miRNA-gene regulatory pairs; more evidence will be needed to determine their important functions.

## 5. Conclusions

In conclusion, the different expression profiles of the transcriptome and miRNA of the two pig groups with diverging backfat thickness (HBFT and LBFT) were described. The target genes regulated by DEMs were predicted, and an integrated analysis of DEMs and DEGs was performed. The results suggested that some miRNAs (for example, mir-13-3p, mir-31-5p, and mir-3477-5p), and genes (including *AQP9*, *DKK3*, *GLYCTK*, and *GLIPR1*) are strong candidates for regulating the accumulation of backfat thickness in pigs. Additionally, we found additional potential miRNAs and genes related to fat deposition. These findings provide a foundation for further studies on the backfat thickness of pigs. Moreover, it is worth noting that the target gene obtained by bioinformatics predictions of miRNAs was related to fat deposition, so further experimental verification should be performed in following work.

## Figures and Tables

**Table 1:** Phenotype information of three full-sibling pigs.

Individual	Backfat Thickness of 6–7 Libs (mm)	Slaughter Weight (kg)
L1	32.36	102.6
H1	48.30	104.2
L2	34.96	102.6
H2	55.21	106.6
L3	32.27	108.6
H3	55.86	107.2

**Table 2:** The function and related miRNAs of candidate differentially expressed genes (DEGs).

Candidate DEGs	Regulation Direction of mRNA (HBFT/LBFT)	Gene Function	Related miRNAs
GLIPR1	up	Protein coding gene, related to regulation of lipid metabolism by peroxisome proliferator-activated receptor alpha (PPARalpha) pathways	mir-13-3p, mir-31-5p, mir-215-5p, mir-3477-5p, mir-200-3p, mir-2-3p, mir-2188-5p
AQP9	up	Encode aquaglyceroporins; facilitate the uptake of glycerol in hepatic tissue	mir-283-5p, mir-31-5p, mir-378-5p, mir-466-5p, mir-71-5p, mir-485-5p, mir-491-5p, mir-215-5p, mir-13-3p, mir-745-3p, mir-995-3p, mir-128-3p, mir-1388-3p, mir-196-5p, mir-3477-5p
SH2B1	up	Encoded protein; mediate activation of various kinases; related to obsity	mir-276-3p, mir-6679-5p
GLYCTK	up	Encoded enzyme; catalyzes the phosphorylation of (R)-glycerate; associated with glycerolipid metabolism	mir-276-3p, mir-485-5p
DKK3	up	Protein coding gene; related to Wnt/β-actin signaling	mir-184-3p, mir-28-5p, mir-3477-5p, mir-378-5p, mir-491-5p
NT5E	up	Encode a plasma membrane protein; associated with backfat thickness by SNP association analysis	mir-210, mir-3477-5p
DUSP2	up	Encode phosphatase; associated with MAPK signaling pathway	mir-184-3p, mir-317-3p, mir-874-5p, ssc-miR-145-5p
CEP19	down	Protein coding gene; linked to morbid obesity	novel-m0104-5p, novel-m0112-5p, novel-m0151-5p
TYSND1	down	Encode protease; related to lipid metabolic; regulate fatty acid β-oxidation	lin-4-5p, mir-140-5p, mir-1692-5p, mir-1895-3p, mir-301-3p, mir-4787-5p, mir-3610-3p, mir-4497-5p, mir-6518-3p, mir-4286, novel-m0065-3p

## Supplementary Materials

The following are available online at https://www.mdpi.com/2076-2615/9/6/313/s1, Figure S1: Heat map of significant differential genes in high backfat thickness and low backfat thickness pigs; Table S1: List of the differentially expressed miRNAs, Table S2: List of the differentially expressed genes; Table S3: The primers of mRNA and miRNA qPCR validation, Table S3: List of the differentially expressed miRNAs, Table S4: Summary of the mRNA sequencing data, and Table S5: List of the differentially expressed genes.
